# Exploring the role of the JEEViKA swasthya mitra helpdesk in improving healthcare access: a qualitative study in tertiary healthcare facilities in Bihar, India

**DOI:** 10.1186/s12913-025-12299-3

**Published:** 2025-01-27

**Authors:** Pragya Kumar, Shamshad Ahmad, Narottam Pradhan, Neha Chaudhary, Haripriya H., Sarita Kumari, Naveen K. G., Akanksha Yadav, Rakesh Jha, Md. Ashraf Parwez, Swati Swati, Apolenarius Purty, Somya Somya

**Affiliations:** 1 All India Institute of Medical Sciences Patna, Patna Bihar, India; 2 Project Concern International India, Patna Bihar, India; 3 ESIC Medical College Bihta, Patna Bihar, India; 4 Saveetha Medical College, Chennai, India; 5 Bihar Rural Livelihoods Promotion Society (BRLPS) “JEEVIKA”, Patna Bihar, India

**Keywords:** Healthcare access, JEEViKA, Patient navigation, Public healthcare systems, Qualitative study, Swasthya Mitra

## Abstract

**Background:**

Rural populations in Bihar, India, face significant healthcare access challenges due to geographical, infrastructural, and financial barriers. The Swasthya Mitra program, initiated by the Bihar Rural Livelihood Promotion Society in collaboration with local and international partners, aims to mitigate these challenges by employing trained community members to navigate patients through the healthcare system.

**Methods:**

This qualitative study employed in-depth interview and thematic analysis to evaluate the Swasthya Mitra program in the Bhagalpur and Jamui districts in Bihar, India. The participants included Swasthya Mitras, healthcare professionals, and beneficiaries. This study focused on understanding the role of Swasthya Mitras in facilitating healthcare services.

**Findings:**

The program improved healthcare access for rural populations, with beneficiaries reporting reduced navigational confusion, decreased out-of-pocket expenditures, and enhanced patient care. Swasthya Mitras bridged the gap between the community and healthcare facilities. The program also empowered women, both Swasthya Mitras and beneficiaries, by improving their access to healthcare and increasing their involvement in health-related decision-making.

**Interpretation:**

The Swasthya Mitra program may offer a viable model for improving healthcare access in rural settings, demonstrating the importance of community involvement in healthcare delivery. The findings suggested that such programs can be instrumental in overcoming barriers to healthcare access and reducing financial burdens on vulnerable populations.

**Funding:**

The study was supported by the Bihar Rural Livelihood Promotion Society supported by the World Bank and Project Concern Internation India.

**Supplementary Information:**

The online version contains supplementary material available at 10.1186/s12913-025-12299-3.

## Introduction

Access to affordable and quality healthcare services is a fundamental right, yet in many rural and underserved areas, individuals face significant challenges in obtaining timely and appropriate medical care [1]. Rural populations often suffer from geographical barriers, limited healthcare infrastructure, and financial constraints, all of which contribute to reduced access to healthcare. These challenges are particularly pronounced in low-resource settings, such as rural regions in low-income countries [2]. The issue of limited healthcare access in rural areas has far-reaching consequences, with individuals often having to travel long distances to seek medical attention [3]. This results in not only delayed care but also increased out-of-pocket expenditures due to the involvement of intermediaries who may direct patients toward costly private practitioners and hospitals. In many cases, these intermediaries exploit the vulnerable situation of patients, worsening the financial burden on already marginalized communities. One approach to addressing these challenges is through patient navigation, a concept introduced in the 1990s to reduce barriers to timely healthcare, particularly for marginalized and underserved populations. Patient navigators are individuals trained to guide patients through complex healthcare systems, ensuring they access appropriate care efficiently and reduce delays in treatment [4]. Initially implemented in cancer care, this model has since expanded to improve access to services for various health conditions, particularly in low-resource settings. Navigators play a critical role in connecting patients to healthcare services, enhancing their understanding of hospital systems, and alleviating systemic burdens such as long waiting times and administrative confusion [5].

In 2006, the Bihar Rural Livelihoods Promotion Society, an independent organization under the Department of Rural Development, began the JEEViKA program with the World Bank. Its main goal is to help underprivileged rural women improve their livelihoods and incomes. Several interventions, including the construction of women-led self-help groups (SHGs), ability building and reinforcement, and bank-SHG financial links, have achieved this goal. These groups have become membership-based social service and corporate organizations. The JEEViKa program has demonstrated considerable success in empowering women by enhancing their economic contributions [6]. In addition to achieving economic empowerment, it acknowledges the significance of health in attaining sustainable development. JEEViKA has implemented numerous interventions within the health sector.

Recent insights from JEEVIKA's self-help group members are a stark indicator of the pressing health challenges faced by communities. Alarmingly, 37% of the loans available to these individuals are funnelled directly into medical expenses, underscoring the substantial economic burden on these self-help groups [6]. The problem at hand was the limited access to healthcare services and the resulting financial burden faced by self-help members due to the involvement of intermediaries and private practitioners. Innovative solutions are needed to improve healthcare access and lower out-of-pocket costs. A Health Help Desk staffed by trained self-Help group members, called "Swasthya Mitra," can help patients navigate the public healthcare system and obtain the right care at the right time. The Swasthya Mitra programme encouraged local communities to advocate for their health and well-being.

JEEViKA, Project Concern International, and All India Institute of Medical Science, Patna trained 217 Swasthya mitra in ten sessions from August 2022 to March 2023 to operate this health help desk. The idea called for patient navigation at all Bihar district hospitals and medical college hospitals. This programme was created to support SHG members and their families during hospitalization for mother–child health emergencies. This support covered medical and non-medical needs. Support was thereafter extended to all patients in the hospital.

This qualitative descriptive study explored the hospital-based "Swasthya Mitra programme" for vulnerable populations in Bhagalpur and Jamui, Bihar, India. The study asked, "How was the Swasthya Mitra programme perceived by beneficiaries and health care professionals on healthcare access and out-of-pocket expenditure?" This study assumed that educating self-help group members as healthcare navigators under the Swasthya Mitra programme would improve healthcare utilization, minimize intermediary reliance, and lower beneficiary out-of-pocket healthcare costs.

## Materials and methods

### Research team and reflexivity

The research team comprised two qualitative research-trained faculty members: PK as the Principal Investigator and SA as the Co-Principal Investigator. The team also included four trained resident researchers: NC, SK, HH, and NKG (three females and one male), all holding MD degrees in Community Medicine. The residents were proficient in regional dialects, with two being Bihar locals—one from the study sites—facilitating effective communication during interviews. The in-depth interviews were conducted by the trained resident researchers, who worked in pairs to ensure thorough data collection and support. For Bhagalpur, one resident conducted the interviews while the other assisted, and similarly for Jamui, another pair took responsibility for data collection. The lead investigators (PK and SA) trained the resident researchers for one week in qualitative research techniques, including informed consent processes, open-ended questioning, and transcription methods. The resident researchers had no prior relationships with the participants, which enabled open and unbiased responses during interviews.

### Study design and theoretical framework

This study employed a qualitative descriptive design to explore healthcare utilization, expenditures, and the perceptions of beneficiaries and stakeholders regarding the Swasthya Mitra program. The study aimed to gain in-depth insights into the program's role in improving healthcare access and delivery to inform healthcare policy and practice. The study followed an interpretivist/constructivist research paradigm. This approach focused on participants' experiences and perceptions to understand the program's contextual effectiveness.

### Participant recruitment and selection

Participants were purposively sampled to ensure representation of key stakeholders involved in or benefitting from the Swasthya Mitra program. A total of 21 participants were recruited from two districts, Bhagalpur (9 participants) and Jamui (12 participants), representing beneficiaries, Swasthya Mitras, and healthcare professionals. The beneficiaries were contacted via phone, and the study's purpose was clearly explained to them. Their interviews were conducted at their homes. In contrast, interviews with Swasthya Mitras and healthcare professionals were conducted at the hospital settings where they worked. All participants provided informed consent, and there were no instances of refusal or dropout throughout the study.

### Data collection

An in-depth interview guide with open-ended questions was developed to capture participants' experiences, challenges, and recommendations (Supplementary File). The guide was pilot-tested and refined based on feedback. Key areas of inquiry included as follows: Swasthya Mitras: Background, motivations, role experiences, obstacles faced, and suggestions for improvement; Healthcare Professionals: Views on Swasthya Mitras’ role, challenges faced by government hospital patients, and recommendations for better integration; Beneficiaries: Perceptions of Swasthya Mitras, experiences with the program, and suggestions for service improvement.

Interviews were conducted in Hindi, audio recorded with participant consent, and transcribed verbatim. Field notes were maintained to capture contextual details. Data saturation was ensured by conducting interviews until no new themes emerged. Interviews lasted between 10 and 26 min, with an average duration of 16 min.

### Data analysis

The data analysis process was iterative and inductive, ensuring a data-driven approach. After transcription and translation into English, all transcripts were imported into NVIVO qualitative data analysis software for systematic coding and management.

The analysis involved a systematic and iterative approach. First-cycle coding applied attribute, descriptive, in vivo, emotion, and process coding techniques to identify initial codes from the transcripts. Two investigators independently coded the data and developed separate initial codebooks. These codebooks were compared, and discrepancies were resolved through thorough discussions, leading to a unified coding framework finalized by consensus. The themes were derived inductively from the data. Patterns and relationships among the codes were then examined, which led to the development of categories and overarching themes that reflected participants' lived experiences and perceptions.

To ensure data quality, the principal investigators and research team iteratively reviewed the transcripts, codes, and emergent themes. Triangulation of data sources, including Swasthya Mitras, healthcare professionals, and beneficiaries, was conducted to enhance the credibility and reliability of the findings. While participant checking could not be performed due to logistical challenges, reflexivity and team consensus were maintained throughout the analysis process to minimize researcher bias and ensure a robust and faithful representation of the data. This study adhered to the Consolidated Criteria for Reporting Qualitative Research (COREQ) guidelines to ensure comprehensive reporting of the methodology and findings [7].

### Ethical considerations

Ethical approval was obtained from the Institutional Ethics Committee at All India Institute of Medical Sciences, Patna (AIIMS/Pat/IEC/2022/1048). Participants were provided with a detailed participant information sheet, outlining the study’s objectives, methods, risks, and benefits. Informed consent was obtained from all participants, and they were assured of confidentiality, anonymity, and their right to withdraw at any point without consequences. Data were securely stored, anonymized, and accessible only to the research team. Audio recordings were destroyed after transcription.

## Results

### Participants characteristics

Table 1 The study included a total of 21 participants across two districts: Bhagalpur (9 participants) and Jamui (12 participants). In Bhagalpur, participants included beneficiaries (3 females, aged 32–38), Swasthya Mitras (3 females, aged 28–37), and healthcare professionals such as a health manager (male, 42), a medical officer (male, 28), and a nursing officer (female, 38). In Jamui, the participants comprised beneficiaries (4 females, aged 24–29), Swasthya Mitras (2 females, aged 28–29), and healthcare professionals, including civil surgeons (male, 55), deputy director of administration (male, 47), medical officers (1 male and 1 female, aged 38 and 36), and nursing officers (2 females, aged 30 and 36).

**Table 1:** Study participants characteristic

District	Designation	Gender	Age
Bhagalpur (9)	Beneficiary (1)	Female	38
	Beneficiary (2)	Female	38
	Beneficiary (3)	Female	32
	Health Manager	Male	42
	Medical Officer	Male	28
	Nursing Officer	Female	38
	Swasthya Mitra (1)	Female	35
	Swasthya Mitra (2)	Female	28
	Swasthya Mitra (3)	Female	37
Jamui (12)	Beneficiary (1)	Female	29
	Beneficiary (2)	Female	24
	Beneficiary (3)	Female	28
	Beneficiary (4)	Female	25
	Civil Surgeon	Male	55
	Deputy Director Administration	Male	47
	Medical Officer (1st)	Male	38
	Medical Officer (2nd)	Female	41
	Nursing Officer (1)	Male	36
	Nursing Officer (2)	Female	30
	Swasthya Mitra (1)	Female	29
	Swasthya Mitra (2)	Female	28

Table 2 presents the key themes and categories that emerged from the qualitative analysis of the Swasthya Mitra program’s impact on public healthcare access and delivery. The results are organized into five major themes, each divided into specific categories.

**Table 2:** Themes and categories highlighting the impact of the Swasthya Mitra program on public healthcare access and delivery

Theme	Categories
1. Unveiling Challenges in Government Hospitals	1.1 Navigational Challenges in Public Hospitals
	1.2 Systemic Barriers to Patient Care
	1.3 Perceptions and Fears of Death
	1.4 Patient Decision-Making and Communication
2. Strengthening Public Health Systems	2.1 Reduction in Treatment Initiation Time
	2.2 Reduction in Out-of-Pocket Expenditures
	2.3 Swasthya Mitra Synergy Enhancing Care
3. Empowering Healthcare Access through Women	3.1 Empowerment through Professional Growth
	3.2 Recognition and Influence in Society
	3.3 Impact on Women’s Autonomy and Healthcare
4. Transforming Patient Preferences	4.1 Shifting Preference to Government Hospitals
	4.2 Patient Satisfaction and Trust
	4.3 Inclusive Support for Marginalized Patients
5. Challenges Faced by Swasthya Mitras	5.1 High Expectations from Beneficiaries
	5.2 Long Working Hours
	5.3 Challenges from Third-Party Brokers
	5.4 Limited Awareness Among Doctors
	5.5 Future Directions for Improvement

### Theme 1: Unveiling challenges in government hospitals/public health facilities

Government hospitals play a vital role in providing healthcare to underserved populations, yet they face significant challenges that impact patient care. Patients often encounter difficulties navigating the hospital infrastructure, leading to delays and heightened stress. Systemic issues, such as long waiting times, limited resources, and overcrowding, and further complicated care delivery. Deeply rooted fears and negative perceptions about tertiary care facilities discourage timely healthcare utilization, particularly in vulnerable communities. Additionally, financial constraints and communication barriers exacerbate patient frustrations, with many struggling to understand diagnoses and treatment instructions.

### Category 1.1: Navigational challenges in public hospitals

Navigational difficulties were identified as a critical barrier to accessing timely healthcare in public hospitals. Patients often struggled to locate service areas within the complex hospital infrastructure, a problem that was particularly acute for elderly patients. Many participants described the lack of clear signage and assistance, leading to delays in receiving care and heightened levels of anxiety. A medical officer highlighted the frequent confusion faced by patients:

*“They do not get to know the exact location where they have to go. For example, many times when we tell them to go to the second floor, they end up standing outside the Operating Theatre (OT).” (Medical Officer-1, DH Jamui).*

For elderly patients, the challenges were even more pronounced. Often navigating hospital premises alone, they were particularly vulnerable to becoming lost or disoriented. One Swasthya Mitra explained:

*“For instance, when elderly people come in, they get lost. No one comes with them… they get distressed.” (Swasthya Mitra-1, JLNMCH)*

Similarly, beneficiaries recounted how the overwhelming nature of the hospital environment exacerbated their distress and confusion. For many, hospital premises, combined with a lack of clear instructions or support, created an intimidating and disorienting experience. Patients unfamiliar with the hospital’s structure often found themselves running from one area to another, unsure of where to go or whom to approach for assistance. One beneficiary described their experience.

*“In a government facility, we have to run here and there a lot because we are totally unaware of the structure and functions of the facility and what should be done where. We were completely clueless.” (Beneficiary-1, JLNMCH Bhagalpur).*

This sense of helplessness was particularly acute for first-time visitors or those with limited literacy, who struggled to navigate the system independently. The disorientation and confusion experienced, particularly by vulnerable groups like the elderly.

### Category 1.2: Systemic barriers to patient care in public hospitals

Public hospitals often serve as the safety net for patients who are unable to access care in private facilities, but this role brings challenges for both patients and the healthcare system. One critical issue highlighted by participants was the difficulty in admitting certain patients, particularly those with complex or critical conditions that private hospitals are unwilling to treat. A health manager explained,

*“Yes, ma’am, private doesn’t admit those kinds of patients. It is an important thing for us; we got her admitted and treated here.” (Health Manager, JLNMCH).”*

Another issue was the long waiting times that patients had to endure in public hospitals, which was a recurring source of frustration for both patients and healthcare providers. This not only delayed medical attention but also created difficulties for individuals who visited hospitals alone. A nursing officer *remarked,*

*“The biggest problem is having to stand in long queues. However, now nobody is helpless. When someone comes alone, they do not get any facility. They do not understand on their own where to go.” (Nursing Officer-1, JLNMCH Bhagalpur).*

Furthermore, the chaotic nature of public hospitals, characterized by overcrowding and insufficient organization, was described as a major deterrent to seeking care.

*“The trouble faced in running around is greater than not getting treated. That is why no one wants to go to a government hospital. Otherwise, there is no other issue.” (Beneficiary-2, JLNMCH Bhagalpur).*

Such chaos not only disrupts the efficiency of healthcare delivery but also discourages many individuals from accessing essential medical services.

Together, these systemic barriers reflected the broader issues within public healthcare systems. These obstacles compromised the overall patient experience, placed undue stress on individuals seeking care, and further strained already overburdened hospitals.

### Category 1.3: Perceptions and fears of death surrounding government health facilities

Healthcare utilization in tertiary facilities was influenced by public sentiment, which was often shaped by myths, rumors, and negative past experiences. Many individuals were deterred from seeking treatment due to deeply ingrained fears and misconceptions about these hospitals. One pervasive belief was that patients admitted to tertiary care facilities rarely survived, causing a perception that entering such a hospital was equivalent to a death sentence. These fears were not only personal but also cultural, passed down through generations, creating a deeply rooted phobia of seeking care in these settings.

*“Earlier, we used to be scared to go to Mayaganj (Medical College) because everyone would say that once you reach Mayaganj, it is the end; you will not come back. That is why we were very scared about it and did not go there. Everyone at home would start crying and wailing.” (Beneficiary-1, JLNMCH, Bhagalpur).*

This fear led to families avoiding tertiary hospitals, even when such facilities were the most appropriate option for their medical needs. These rumors and inherited anxieties created a substantial emotional barrier, preventing individuals from accessing potentially life-saving medical interventions. The widespread apprehension not only delayed timely care but also contributed to the worsening of health conditions, further perpetuating the cycle of mistrust and fear surrounding tertiary care facilities.

### Category 1.4: Patient decision-making and communication barriers in healthcare

Patient decision-making in healthcare was deeply influenced by their financial capacity and the perceived quality of care, with many patients favoring private facilities when resources allowed. This preference often stemmed from the belief that private doctors were more attentive and accessible compared to their counterparts in public hospitals. A medical officer explained,

*“I sit in my OPD in the government hospital; the patient starts asking when I will sit in my private clinic. Even if I charge them with fees (it is free in the government hospital), they will come there. I cannot change this mentality.” (Medical Officer-2, District Hospital Jamui).*

Another beneficiary highlighted the financial dimension of these decisions, stating,

*“If we have money, we go to a private health facility.” (Beneficiary-2, District Hospital Jamui).*

Compounding this issue were communication barriers within public health facilities. Many patients, particularly those with limited education, struggled to comprehend their diagnoses, treatment plans, or posttreatment instructions. Language difficulties and a lack of clear, patient-centered communication from healthcare providers often left patients confused and uninformed about their care. A resident explained,

*“Most of the patients and their attendees here are not well educated. Therefore, they face problems understanding doctors’ advice.” (Medical Officer, JLNMCH Bhagalpur).*

This communication gap frequently led to poor adherence to follow-up protocols and post-treatment care, creating a cycle of inadequate healthcare utilization. Patients’ inability to fully grasp medical guidance not only affected their immediate health outcomes but also contributed to long-term challenges in managing chronic conditions or understanding preventive measures.

### Theme 2. Strengthening public health systems with Swasthya Mitra

Swasthya Mitra role in guiding patients through hospital processes has reduced treatment initiation time, ensuring that patients receive medical attention promptly, especially during critical situations. By managing administrative tasks and providing directions, they have eased the workload of hospital staff, allowing them to focus more on clinical responsibilities. In addition, Swasthya Mitras have helped patients navigate the public healthcare system effectively, reducing financial burdens by steering them toward cost-effective services and avoiding unnecessary expenditures. Their ability to coordinate care across departments and assist patients with procedures has streamlined hospital operations, making healthcare delivery more organized and accessible. Through these efforts, Swasthya Mitras have contributed to improving both the efficiency and responsiveness of public health systems.

### Category 2.1: Reduction in the time of treatment initiation

The proactive involvement of Swasthya Mitras played a role in expediting the process of treatment initiation, ensuring that patients receive timely care. By guiding patients through hospital protocols, assisting with necessary paperwork, and providing reassurance, Swasthya Mitras reduced the delays that patients often faced upon entering the healthcare system. Their presence alleviated the initial confusion and stress experienced by patients, particularly those unfamiliar with hospital settings or facing medical emergencies.

One beneficiary described their experience of being overwhelmed upon arriving at the hospital:

*“We called up. We said, ‘Where will we go?” We do not even know.’ Sister (Swasthya Mitra) said, ‘Come to me, I will be at the gate.’ At first, we were very anxious. She calmed us down. She said, ‘You do not have to worry. Now that you are here, everything will be taken care of.’ After that, she got us a token, and then she took us to the doctor.” (Beneficiary-3, JLNMCH, Bhagalpur).*

This immediate intervention not only provided emotional support but also eliminated time-consuming hurdles that often delay access to medical attention.

Swasthya Mitras were particularly effective in managing critical cases, such as labor and delivery emergencies. Their ability to promptly direct patients through essential investigations and facilitate rapid transfers to the labor room was highly valued by medical teams. A medical officer explained,

*“Many times, when a patient comes into labor, the Swasthya Mitra directly brings them in and guides them through the necessary investigations. She ensures that the patient is swiftly moved to the labor room. This saves time for the medical team and benefits the patient, as their management and care can begin more quickly. (Medical Officer-2, District Hospital, Jamui).*

By reducing the time from hospital entry to treatment initiation, Swasthya Mitras ensured that patients received medical attention with minimal delays, which was particularly crucial in emergency cases.

### Category 2.2: Reduction in out-of-pocket expenditure and financial barriers

In a healthcare system where costs can escalate rapidly, finding a financially viable solution was crucial for many patients. Public hospitals, with the support of Swasthya Mitras, emerged as a lifeline for individuals facing financial and emotional burdens during medical emergencies. Swasthya Mitras not only helped patients navigate the complexities of the public healthcare system but also played a pivotal role in reducing out-of-pocket expenditures by steering them toward cost-effective treatment options.

One beneficiary shared a critical moment during her pregnancy when a private doctor recommended immediate surgery due to complications, warning that delays could endanger the baby’s life. Overwhelmed by the financial implications, the family remembered the guidance provided by the Swasthya Mitra and chose a government hospital instead.

*“When it was time for delivery, we went to one doctor in private. There, we were told that the baby's umbilical cord was wrapped around its neck, and if surgery was not performed within half an hour, the baby would not be able to be saved. We remembered what our sister had said during a meeting. Therefore, we believe that this approach would be expensive and perhaps not possible. Even, if possible, it would be difficult. Therefore, we went to a government hospital instead.” (Beneficiary-2, JLNMCH, Bhagalpur).*

Swasthya Mitras also provided critical support in cases of accidental injuries, where families often faced financial and logistical challenges. One Swasthya Mitra explained how they reassured families about the affordability of treatment in government hospitals while ensuring that patients receive timely care.

*“Certainly, there has been a difference. For instance, when there is an accidental case, it can cause considerable trouble. Going outside [to other medical facilities] would cost approximately 50–60,000 rupees. Here (the government hospital), your work is done in just 5,000 rupees. Therefore, we explain to them, ‘Brother, you are poor; you might have to wait a bit, but we will get your work done. We will talk to the doctor on your behalf. However, if you still wish to leave or know [about other options], that is your choice. However, let me assure you that your work will be done here easily.’” (Swasthya Mitra-3, JLNMCH, Bhagalpur).*

By providing emotional reassurance and practical guidance, Swasthya Mitras empowered patients to make informed decisions during stressful situations. They became advocates for equitable healthcare access, ensuring that financial constraints did not prevent patients from receiving essential medical care.

### Category 2.3 Swasthya Mitra synergy enhancing patient care

#### Subcategory 2.3.1: Navigation and coordination of care

Swasthya Mitras played a role in guiding patients through the increasingly complex healthcare landscape. They filled a critical gap in hospital navigation, offering directions, information, and ensuring smooth coordination across departments. Patients and staff alike acknowledged their importance in improving the efficiency of care delivery. A nursing officer shared,

*“No, it was not like this before. Ever since Jeevika’s sister came, people do not have to wait. Earlier, people had to determine where to go. Now, she tells them where to consult depending on their needs and even gets the slip for them.” (Nursing Officer-1, DH, Jamui).*

Swasthya Mitras coordinated services such as ambulance dispatch and patient referrals, which were previously challenging for families to manage. Their involvement ensured that patients requiring urgent care were transported promptly and directed to the appropriate departments without unnecessary delays. By maintaining direct communication with ambulance drivers and hospital staff, they streamlined the referral process, reducing the chaos often associated with emergency situations.

*“We have also tied up ambulance services in this manner with them [Swasthya Mitras], which are registered. Therefore, you will see that if a patient needs an ambulance, even the ambulance register is available with them.” (DDA, DH, Jamui).*

By taking on these logistical responsibilities, Swasthya Mitras ensured a level of care coordination that was previously lacking, making healthcare facilities more accessible and responsive to patient needs. Their engagement reduced delays in critical situations and minimized the stress faced by families during emergencies.

#### Subcategory 2.3.2: Bridging communication gaps

Swasthya Mitras served as a vital communication bridge between patients and healthcare providers. For many patients, especially those with limited literacy or understanding of medical terminology, Swasthya Mitras simplified processes, explained treatment steps, and clarified follow-up protocols. Swasthya Mitra recounted,

*“After I made the call, the patient asked, ‘Where should we go? We have no idea.’ I told her to come to me, and we would be waiting at the gate. We took her to the registration counter, then to the doctor.” (Swasthya Mitra, JLNMCH Bhagalpur).*

Their understanding of medical terminology appeared to enhance communication between doctors and patients, potentially improving the flow of information within the hospital. In some instances, Swasthya Mitras seemed to simplify complex medical information and conveyed it to patients and their families in a manner that was easier to understand. This may have helped bridge gaps in communication, allowing doctors to focus on clinical tasks without the added burden of repeated explanations. Swasthya Mitras appeared to take on a facilitative role, ensuring patients were better informed about their treatment plans, follow-up instructions, and necessary administrative procedures.

One Swasthya Mitra shared*,*

*“Doctors explain everything to us first. They only call the attendees to sign.” (Swasthya Mitra-2, JLNMCH Bhagalpur).*

This suggests that doctors relied on Swasthya Mitras to communicate critical information, indirectly enhancing patients' understanding.

By acting as a potential bridge between medical professionals and patients, Swathya Mitras may have alleviated some of the communication challenges within the hospital. Their involvement seemed to provide patients with greater reassurance and clarity about their healthcare journey, contributing to a more supportive experience overall.

#### Subcategory 2.3.3: Compassionate care and follow-up

The compassionate and patient-centered approach of Swasthya Mitras enhanced the care experience. Their ability to prioritize urgent cases, such as labor and delivery emergencies, ensured timely medical interventions. A district health authority official remarked,

*“They know which people need to be seen immediately in an emergency… a sideline opens up for them… they get emergency services a bit faster.” (DDA, Jamui).*

Postoperative and post discharge follow-ups appeared to be an important aspect of the support provided by Swasthya Mitras, potentially enhancing the continuity of care for patients. By maintaining follow-up registers, Swasthya Mitras seemed to take an active role in monitoring the recovery process of patients and addressing any complications that arose. Their efforts may have helped bridge the gap between hospital care and home recovery, ensuring that patients receive consistent support even after leaving the hospital.

A swasthya mitra described this process,

*“After she got discharged, we call her after two days and ask, ‘Sister, how is your cesarean? Are there any issues with the stitches? Is there pain?’” (Swasthya Mitra-1, JLNMCH, Bhagalpur).*

This engagement has reassured patients that their recovery was being monitored, providing them with the confidence to raise any concerns.

In addition to addressing medical issues, Swasthya Mitras appeared to play a role in alleviating the emotional burden of recovery, particularly for patients who lacked strong family support or faced challenges in accessing follow-up care. Their regular contact has encouraged patients to adhere to medical advice and fostered a sense of connection and trust. This approach could have contributed to smoother transitions from hospital to home, potentially reducing the likelihood of complications and remissions while promoting a more positive healthcare experience for patients and their families.

Their warm and friendly behavior may have played a key role in alleviating patient anxiety, particularly for those who were unfamiliar with the system or feeling vulnerable due to their medical conditions.

A medical officer observed,

*“The first thing is that they have a good and smiling face.” (Medical Officer-2, Jamui).*

This simple yet impactful quality likely made patients feel valued and cared for, contributing to a more humane aspect of healthcare delivery. Their role extended beyond navigation and logistics, adding a layer of emotional support that was particularly meaningful in the context of high-stress public healthcare facilities.

### Theme 3: Empowering healthcare access through women’s empowerment

The Swasthya Mitra program also empowered women to take on meaningful roles within the healthcare system. By providing opportunities for professional growth, the program enabled Swasthya Mitras to develop new skills and gain confidence as key facilitators in hospitals. Their contributions earned them recognition and respect within their families and communities, elevating their social influence and reinforcing their value. Moreover, the program fostered women’s autonomy, enabling them to make independent healthcare decisions and advocate for better access to services. The Swasthya Mitra cadre, an extension of JEEViKA’s initiatives, exemplifies this empowerment by equipping women with the skills and confidence to transform healthcare access in their communities.

### Category 3.1: Empowerment through professional growth and versatility

The transition to the role of Swasthya Mitra allowed women to evolve professionally while embracing diverse responsibilities within hospital systems. Many Swasthya Mitras recounted how their roles provided them with platforms to channel their expertise and respond to the needs of those in distress. One Swasthya Mitra shared,

*“In Jeevika, I was working, but there were moments like when a baby died after delivery. In another instance, a woman had low blood levels after childbirth. Despite teaching and advice, we could not provide effective help.” (Swasthya Mitra-1, JLNMCH Bhagalpur).*

She reflected that becoming a Swasthya Mitra empowered her to directly address such challenges and ensure patients receive timely care. This professional transformation also expanded their skill sets, making them indispensable contributors to healthcare delivery. Their ability to handle multiple responsibilities earned them the title of *"all-rounders."* As one Swasthya Mitra explained,

*“We are all-rounders in the hospital because while a nurse or a sister might specialize in one area, we have knowledge about many fields and departments.” (Swasthya Mitra-3, JLNMCH Bhagalpur).*

This versatility not only enhanced their confidence but also demonstrated the value of their multifaceted roles in improving healthcare outcomes.

### Category 3.2: Recognition and influence in family and society

The work of Swasthya Mitras extended beyond hospital walls, positively impacting their families and communities. Their roles allowed them to acquire medical and communication skills that gained the respect and admiration within their social circles. Swasthya Mitras described how their newfound knowledge, including familiarity with technical medical terminology and procedures, elevated their standing among family members. One Swasthya Mitra noted,

*“Since we started working here, we have gained a lot of experience. During training, we were not informed about the details of the investigations that doctors wrote, but as we started working, we learned a lot of things.” (Swasthya Mitra-1, JLNMCH Bhagalpur).*

Family members often recognized and appreciated the professional growth of Swasthya Mitras, complimenting them on their ability to navigate complex medical systems. This acknowledgment not only boosted their self-esteem but also reinforced their influence within family and social settings, further empowering them to advocate for health and well-being.

### Category 3.3: Impact on women’s autonomy and healthcare access

The Swasthya Mitra cadre exemplifies how empowering women can lead to transformative changes in healthcare accessibility. Through their work, Swasthya Mitras were not only able to support patients but also became role models for other women in their communities, showcasing the possibilities of economic and social independence. Their engagement in health and nutrition initiatives bridged critical gaps in community healthcare, addressing issues that disproportionately affected women and children.

By confidently interacting with doctors, assisting patients, and advocating for better care, Swasthya Mitras enhanced their own autonomy while improving healthcare access for marginalized groups. As one Swasthya Mitra expressed,

*“Living in the village, we interacted with people. Now we converse with prominent doctors. If patients struggle to communicate, we can confidently bridge the gap. When doctors cooperate, we feel empowered and capable.” (Swasthya Mitra-2, JLNMCH Bhagalpur).*

The program’s emphasis on women-led empowerment created ripple effects, enabling Swasthya Mitras to serve as both caregivers and catalysts for social change. Their efforts not only brought healthcare closer to those in need but also redefined the role of women in community health, emphasizing the potential of inclusive and community-driven healthcare models.

### Theme 4: Transforming patient preferences and collaborative satisfaction

The Swasthya Mitra program played a pivotal role in reshaping patient perceptions and preferences regarding public healthcare facilities. By addressing key barriers to access, the program encouraged a noticeable shift from private to government hospitals, where patients found greater affordability and support. Swasthya Mitras enhanced trust and satisfaction among patients by providing compassionate care, clear guidance, and emotional reassurance in often overwhelming hospital environments. Additionally, the program ensured inclusive support for marginalized and underserved populations, who might otherwise struggle to access necessary healthcare services.

### Category 4.1: Shifting preference from private to government hospital

The introduction of the Swasthya Mitra cadre appeared to drive a noticeable shift in patient preferences from private healthcare facilities to government hospitals. Previously, patients often favored private facilities, perceiving them as more organized and accessible. However, with the support of Swasthya Mitras, government healthcare services became more approachable, reducing confusion and financial barriers for patients. This shift was particularly for individuals from marginalized communities, who began to see public healthcare as a viable and cost-effective option. Patients described how the presence of Swasthya Mitras alleviated the stress of navigating government hospitals, which were often seen as overwhelming and disorganized. One beneficiary recounted,

*“In a government facility, we have to run here and there a lot because we were totally unaware of the structure and functions of the facility and what should be done where. We were completely clueless, but after the Swasthya Mitra cadre was created, she used to explain to us what should be done and where we should go. It was quite easy to manage our health needs in the government facility itself without spending a lot of money from our pocket.” (Beneficiary-3, JLNMCH, Bhagalpur).*

The presence of Swasthya Mitras seemed to act as a bridge, transforming perceptions about public healthcare and enhancing trust and reliance on government facilities. This shift highlights the potential of patient navigation programs to enhance the usability and attractiveness of public health systems, ensuring that essential services are not just available but also approachable and effective.

### Category 4.2: Patient satisfaction and trust

The presence of Swasthya Mitras in government hospitals appeared to enhance a notable improvement in patient satisfaction and trust in the healthcare system. Patients often highlighted how Swasthya Mitras treated them with a level of compassion and attentiveness that was not commonly experienced in busy public hospitals. Unlike the often-transactional interactions with regular hospital staff, patients described the care and assistance provided by Swasthya Mitras as personalized and empathetic, making them feel valued and supported during stressful times.

One beneficiary reflected on the difference, saying,

*“No one in such a big hospital talks to us the way they (Swasthya Mitra) talked. Nurses here do not talk nicely when giving injections; they say things like 'your arm is swollen, extend it properly' (strict tone).” (Beneficiary-2, JLNMCH, Bhagalpur).*

In contrast, Swasthya Mitras offered patients a listening ear, clear explanations, and a gentle demeanor, giving a sense of trust and comfort even in challenging circumstances. The emotional support provided by Swasthya Mitras also appeared to play a role in addressing the anxiety and fear that patients often felt in unfamiliar hospital settings. Their ability to provide timely assistance and explain hospital processes in a patient-friendly manner ensured that individuals felt more informed and in control of their care.

Moreover, healthcare staff also acknowledged the impact of Swasthya Mitras on improving patient satisfaction. By bridging communication gaps, providing emotional reassurance, and reducing the chaos often associated with public healthcare settings, Swasthya Mitras created a more patient-centered environment.

### Category 4.3: Inclusive support

The Swasthya Mitra program appeared to provide critical support for marginalized patients, particularly those facing social stigma or financial challenges. By offering inclusive assistance, Swasthya Mitras helped individuals navigate the barriers often encountered in both private and public healthcare systems. One particularly compelling example involved a local HIV-positive patient who was initially denied care at a private facility. Her family, struggling with limited financial means, faced obstacles in accessing essential medical services until they sought the help of a Swasthya Mitra.

A health manager recounted the case:

*“There was a local patient from a nearby place; she was HIV positive. Her husband was a rickshaw puller. Initially, they visited a private facility, but they were not admitted due to her HIV status. Later, they heard about Didi (referring to Swasthya Mitra) and contacted her. She helped, and the patient was admitted. The delivery was successful, and the baby was healthy. This effort is truly commendable.” (Health Manager, JLNMCH).*

The inclusive approach of the Swasthya Mitras extended beyond individual cases, as they addressed systemic challenges that disproportionately affected disadvantaged populations. Their ability to bridge gaps in care, particularly for those overlooked or underserved by traditional healthcare models, garnered widespread appreciation from both patients and healthcare providers.

### Theme 5: Challenges faced by Swasthya Mitras and future directions

While the Swasthya Mitra program has improved healthcare access and delivery, the cadre faced considerable challenges in their roles. High expectations from beneficiaries often exceeded their scope and available resources, creating operational strain. Long working hours, coupled with increasing patient demands, added to their workload and fatigue. Interference from third-party brokers posed additional difficulties, sometimes leading to conflicts and safety concerns. Furthermore, limited awareness among doctors about the Swasthya Mitras' roles hindered effective collaboration within the healthcare system. Despite these obstacles, the program holds promise for improvement through better integration, enhanced training, and supportive measures to strengthen its sustainability and impact.

Swasthya Mitras played a crucial role in bridging the gap between patients and healthcare services in government facilities. However, their work was not without challenges. They grapple with high patient expectations, resource limitations, administrative pressures, and the need for better infrastructure. The initial phase of work for Swasthya Mitras was arduous. Patients, sometimes with high hopes, sought assistance, assuming that Swasthya Mitras would take care of their concerns. This created expectations that challenged the limited resources and capabilities of these healthcare facilitators.

*"In the beginning, we faced many difficulties. For instance, when we started out, any patient who would come were optimistic, thinking 'Didi (sister) will get all my work done. (Swasthya Mitra-1, JLNMCH, Bhagalpur).*

### Category 5.1: High expectations from beneficiaries

Complexities arose when patients required timely operations, and delays occurred due to resource constraints. Patients and their families experienced frustration when operations did not occur as expected, highlighting the strain on healthcare services.

*"Therefore, for instance, when we got the patient admitted to ORTHO (orthopedic department), their surgery should have taken place after 2–3 days. However, the surgery did not occur. Everyone became very worried, thinking, 'It has been 4 days, and our surgery hasn’t been done. '" (Swasthya Mitra-3. JLNMCH, Bhagalpur).*

### Category 5.2: Long working hours

Swasthya Mitras described their demanding routines, involving long shifts and continuous availability to attend to patients. As patient footfalls increase throughout the day, maintaining patient records becomes challenging, often leading to incomplete documentation. Patients sometimes misinterpreted or misused the role of Swasthya Mitras, asking them to complete administrative tasks such as issuing certificates.

*"We have to do an 8-h duty. We arrived at 6 AM in the morning. After that, the phone becomes busy, so we must answer it. Currently, token issuing (registration) starts at 8 AM, so patients start coming in at 8 AM, and we get busy assisting them. Then, we must maintain the register, and sometimes there are issues with it, which causes delays. We were unable to complete all the columns for every patient. Therefore, we finish it on the second day."( Swastya Mitra-2, Jamui).*

### Category 5.3: Challenges faced by Swasthya Mitra due to third-party brokers

One of the most challenges faced by Swasthya Mitras stemmed from the interference of third-party brokers, commonly referred to as "dalals." These brokers frequently sought to exploit vulnerable patients for personal financial gain, often compromising patient welfare and undermining the efforts of the Swasthya Mitra program. Their actions created substantial obstacles in areas such as ambulance services and patient referrals, while also fostering a hostile environment for Swasthya Mitras themselves.

The brokers often attempted to intercept ambulances arriving at hospitals, diverting patients to private practitioners or facilities that were more expensive and often unnecessary. This misdirection led to delays in care and increased financial burdens for families. Swasthya Mitras, working closely with ambulance drivers, made efforts to counter these actions by ensuring patients were brought directly to the hospital without interference. As one Swasthya Mitra described,

*“Ever since we got the charge of ambulance facility management, we get referrals from PHCs (primary health centers). The referrals that come here, what those people (brokers) do is stop the ambulance right there (outside the hospital gate). They divert the ambulance to private hospitals. Ever since we got the ambulance, the drivers tell us, 'Sister, patients are coming. Please take care of them; otherwise, they will fall into the hands of the agents.’” (Swasthya Mitra-1, Jamui).*

In addition to interference with patient referrals, brokers often resorted to verbal abuse and threats to intimidate Swasthya Mitras. In some instances, these threats escalated to physical confrontations, creating an unsafe and hostile working environment. One Swasthya Mitra recounted,

*“Those agents were saying that ever since Jivika didi took charge, their earnings had virtually stopped. One day, they were verbally abused even as they walked by. They even tried to get into a physical altercation with us, saying that one day you will get beaten up if you keep doing this.” (Swasthya Mitra-2, Jamui).*

These challenges not only jeopardized the safety of Swasthya Mitras but also hindered their ability to perform their roles effectively. Despite these difficulties, Swasthya Mitras remained committed to their mission of improving patient access to affordable and timely care, demonstrating resilience and dedication in the face of adversity.

### Category 5.4: Limited awareness of Swasthya Mitras’ role among doctors

While Swasthya Mitras played a vital role in enhancing patient care and experience, their presence within hospitals often went unrecognized or misunderstood by doctors. There appeared to be a gap in awareness regarding the purpose and responsibilities of Swasthya Mitras among medical professionals, which may have limited their ability to collaborate effectively.

Doctors frequently expressed uncertainty about the specific functions of Swasthya Mitras within the hospital environment.

*“I have no idea. They might help direct patients to the right place, such as an enquiry desk.” (Medical officer, Bhagalpur).*

This lack of clarity reflected the absence of formal communication channels or introductions that could have helped integrate Swasthya Mitras more seamlessly into the hospital's operations.

Some doctors assumed that Swasthya Mitras were merely extensions of hospital administration rather than independent facilitators with a defined role in patient navigation.

*“We maximum doctors thought they were on behalf of hospital administration.” (Medical Officer, Bhagalpur).*

Although doctors acknowledged the positive interactions between Swasthya Mitras and patients, they highlighted the lack of direct engagement or formal collaboration between themselves and the cadre. One doctor reflected,

*“Yeah. I admit they accompany patients to OPDs, but still we were clueless, 'who are they?' The interaction between SM and patients seems to be great. However, no interaction/introduction was given by SM to doctors.” (Medical Officer, Bhagalpur).*

This gap in communication and understanding might have impeded the development of collaborative relationships, potentially limiting the full impact of Swasthya Mitras on patient care.

### Category 5.5: Future directions for enhancing Swasthya Mitras’ impact

Several key areas for improvement were identified to enhance the effectiveness and reach of the Swasthya Mitra program. These recommendations focussed on addressing operational challenges, increasing visibility, and strengthening their integration within the hospital system.

One suggestion involved the introduction of printed templates or guides detailing hospital services and respective room numbers to improve navigation for patients and their families. These materials could reduce the time and confusion associated with locating departments or services.

*“If we could get a printed template-type guide of where and what services are available in the hospital, then when explaining to someone, we could also give them a piece of paper. If the printed services are all laid out in an organized manner detailing all the arrangements in the hospital, then we can give them directly in written form.” (DDA, Jamui).*

Increasing the visibility of Swasthya Mitras was another area of focus. Recognizable uniforms or badges could help patients identify them easily, while regular training sessions were suggested to ensure they stay updated with the latest healthcare protocols and practices. A civil surgeon highlighted the success of self-help group-led initiatives like Didi ki Rasoi (an initiative by JEEVIKA members to start and maintain a hospital kitchen) and suggested a similar model for expanding the roles of Swasthya Mitras:

*“Today, there is ‘Didi ki Rasoi,’ fully operated by them (JEEVIKA self help group). The way and time it have been set up, a place has been created in a hospital where one can sit and eat comfortably. Similarly, Swasthya Mitras will need to be added a bit more, and this will happen.” (Civil Surgeon, Jamui District).*

Refresher training was emphasized as crucial for maintaining the skills and readiness of Swasthya Mitras. A senior official explained,

*“In the initial stages, it is like looking at a plot. In the field, if a plow is used, it runs smoothly for the first time. However, unless it is run a couple of times more, it does not get perfectly ready. These individuals are also completely unfamiliar with the medical field. Hence, you will need to provide them with fresher training time to time.” (DDA, Jamui).*

Availability was identified as a critical issue, particularly during nighttime hours. The absence of Swasthya Mitras at night created opportunities for third-party brokers to exploit vulnerable patients. A health manager observed,

*“They (brokers) are more active at night. Therefore, if the Swasthya Mitras are present at night as well, then the patient will not suffer.” (Health Manager, Bhagalpur).*

Additionally, the current staffing model, where only one Swasthya Mitra per shift was available, sometimes left service counters unattended when they accompanied patients. Having at least two Swasthya Mitras per shift was suggested to ensure uninterrupted service while also assisting patients effectively.

## Discussion

The study explored the Swasthya Mitra program's impact on improving healthcare access and delivery in public hospitals in Bihar, India. Participants, including Swasthya Mitras, healthcare professionals, and beneficiaries, highlighted systemic challenges such as navigational confusion, long waiting times, and communication barriers that hindered patient care. The findings suggested that the program addressed these challenges by improving patient navigation, expediting treatment initiation, and reducing out-of-pocket expenditures. Swasthya Mitras also bridged gaps in postoperative care and offered emotional reassurance, enhancing patients’ confidence in public healthcare facilities. However, challenges, including high patient expectations, third-party interference, and limited integration with hospital staff, persist, requiring targeted interventions to optimize the program’s long-term success.

The study reaffirmed well-documented issues in public healthcare systems, including navigational confusion, delays in care, and communication barriers which were widely reported in global studies. For instance, Teng et al. [8] emphasized that navigational difficulties were exacerbated in complex public health settings, necessitating tailored support to improve healthcare access. Similarly, Qua et al [9] highlighted how structural inefficiencies, such as lengthy waiting times and fragmented care, deterred patients from fully utilizing public health services. Patients described the disorientation they experienced due to inadequate signage and lack of assistance, which was particularly distressing for elderly or first-time visitors. Prior studies, including Schwarz et al. [10] and Boro et al. [11], have similarly identified the need for patient-centric navigation systems to alleviate such confusion. Swasthya Mitras effectively filled this gap by guiding patients, minimizing stress, and reducing delays. The emotional support provided during such instances mirrors findings from Ferrante et al. [12]., who highlighted patient navigators’ role in easing patient anxieties while ensuring timely care. The findings of Dimitropoulos et al. [13] also emphasized the importance of systematic navigation support in improving healthcare access, particularly for vulnerable populations struggling to navigate complex systems. Fillion et al. [5] demonstrated similar results in cancer care settings, where patient navigators reduced barriers, facilitated communication, and improved overall patient satisfaction.

The lengthy waiting times observed in the current study resonate with the findings of Madula et al. [14], who emphasized that resource constraints and inefficiencies exacerbate delays in public hospitals. Patients often faced additional challenges when unaccompanied, leading to frustration and disillusionment with public systems. Swasthya Mitras appeared to mitigate these issues by offering personalized assistance, ensuring smoother patient transitions through hospital services.

Swasthya Mitras played a pivotal role in reducing treatment initiation times, particularly during critical cases such as labor and delivery emergencies. By guiding patients, facilitating paperwork, and coordinating across departments, Swasthya Mitras helped streamline hospital workflows. This aligns with the findings of Basu et al [15]., where navigation systems reduced waiting times, particularly for vulnerable populations. Their ability to coordinate ambulance services and ensure prompt referrals was particularly noteworthy, addressing a key gap in emergency care management.

In addition to logistical support, Swasthya Mitras helped reduce out-of-pocket expenditures by directing patients toward cost-effective treatments within government hospitals. This finding is reinforced by a study conducted by Larsson et al. [16], who reported that navigation programs improved access to affordable care, particularly among marginalized populations. Similarly, beneficiaries in this study recounted instances where timely guidance enabled them to avoid unnecessary financial strain, illustrating the program’s role in enhancing healthcare affordability.

The study highlights how the Swasthya Mitra program contributed to women’s empowerment by offering professional growth opportunities and recognition within their families and communities. Swasthya Mitras described their transformation from community-based workers to all-rounders capable of navigating hospital systems, coordinating care, and bridging communication gaps. These findings align with Toal-Sullivan et al. [17] and Pratt et al. [18], who demonstrated how such initiatives not only enhance healthcare delivery but also empower women as agents of change. By gaining medical and communication skills, Swasthya Mitras earned respect within their social circles, reinforcing their role as healthcare advocates.

The Swasthya Mitra program also appears to have played a crucial role in shifting patient preferences from private to public healthcare facilities. Patients, previously deterred by the perceived disorganization and inefficiencies of government hospitals, expressed greater confidence in accessing care due to the guidance provided by Swasthya Mitras. This shift mirrors findings from Urbanowicz et al. [19], where navigators helped demystify healthcare systems, enhancing trust and satisfaction among beneficiaries. Similar observations were made by Qua et al [9], who reported that navigators’ presence in healthcare facilities mitigated barriers and improved patient trust, particularly among marginalized groups.

Participants further praised Swasthya Mitras’ compassionate demeanor and emotional support, which were not commonly experienced with regular hospital staff. These qualities helped to create patient-centered healthcare environment, consistent with the findings of Esperat et al. [20], who emphasized the impact of culturally sensitive navigation programs on patient satisfaction and adherence to care. Nevertheless, challenges such as limited night-time availability of Swasthya Mitras remain a concern, as identified in the present study.

Despite its successes, the program faced operational hurdles such as high patient expectations, interference from third-party brokers, and gaps in collaboration with hospital staff. Similar challenges were reported by Loo et al. [21], where limited staffing and operational constraints impacted program efficiency. Addressing these challenges requires a multifaceted approach, including improved visibility and infrastructure, developing printed or digital navigation guides to help patients locate services and departments efficiently, and ensuring regular training and skill development. Offering refresher courses will ensure Swasthya Mitras remain equipped to address evolving healthcare needs. Sadak et al. [22] and Thackrah et al. [23] similarly emphasized the importance of equipping navigators with tools and skills to meet evolving patient needs. Strengthening collaboration through regular interactions between Swasthya Mitras, doctors, and hospital staff will enhance mutual understanding and better patient outcomes. Addressing night-time availability by introducing multiple shifts or additional staff could ensure continuous support, particularly during critical hours.

In conclusion, the Swasthya Mitra program demonstrates the potential of patient navigation models in addressing systemic healthcare barriers, improving access, and improving trust in public health systems. By leveraging patient-centered approaches, the program not only improved healthcare experiences but also empowered women as key facilitators of change within their communities. Future efforts should focus on addressing operational challenges and institutional collaboration to ensure the program’s sustainability and scalability, making it a replicable model for strengthening public healthcare systems in resource-limited settings.

## Limitations

The study has few limitations. First, there is a potential for bias as AIIMS Patna was responsible for both training the Swasthya Mitras and overseeing the program. This dual role may have influenced the objectivity of the assessment and the interpretation of the program’s effectiveness and outcomes.

Second, the findings of this study may have limited generalizability to other settings. The context-specific nature of the Swasthya Mitra program, implemented within Bihar's public health system, means that its applicability to other regions with differing healthcare infrastructures, resource availability, and sociocultural contexts may be constrained.

Third, the presence of the Hawthorne effect cannot be ruled out. The involvement of Swasthya Mitras may have temporarily influenced healthcare staff behavior and patient interactions, potentially inflating the observed benefits of the program. Future studies with long-term observations are needed to assess the sustainability of these impacts.

Additionally, the study primarily relied on qualitative data, which, while rich in context and detail, may not fully capture quantifiable outcomes such as treatment success rates or reductions in wait times. Incorporating mixed method approaches in future research could help validate the qualitative findings and provide a more comprehensive understanding of the program's impact.

Lastly, operational challenges, such as staff shortages and resource limitations, may have influenced the performance and effectiveness of the Swasthya Mitras. Addressing these issues remains essential for scaling and replicating the program in similar healthcare settings.

## Supplementary Information

Supplementary Material 1.

Supplementary Material 2.

## Data Availability

The datasets generated and/or analyzed during the current study are not publicly available due to privacy concerns and confidentiality agreements, but are available from the corresponding author on reasonable request. The data supporting the results reported in this article are preserved in a secure environment and can be accessed in accordance with the ethical guidelines and approvals governing this research. Requests for access to these data should be directed to Dr Shamshad Ahmad, drshamshadahmad@aiimspatna.org.

## Abbreviations

JEEViKA: Bihar Rural Livelihood Promotion Society
SHGs: Self-Help Groups
AIIMS: All India Institute of Medical Sciences
NCD: Non-Communicable Diseases
MNH: Maternal and Neonatal Health
