# Supplementary material for: Exploring the role of the JEEViKA swasthya mitra helpdesk in improving healthcare access: a qualitative study in tertiary healthcare facilities in Bihar, India
Source: BMC Health Serv Res. 2025 Jan 27;25:146. doi: 10.1186/s12913-025-12299-3 (PMC11771127; doi:10.1186/s12913-025-12299-3)
Supplement: Supplementary file 2 — Supplementary Material 2. [file 12913_2025_12299_MOESM2_ESM.pdf]

Interview Guide for the Swasthya Mitra Project

Swasthya Mitra Interview Guide

Introduction

Could you introduce yourself? Please share your name, educational background, the Self-Help Group (SHG) and Village Organization (VO) you are associated with, and your location.

Motivation and Experience

What motivated you to join the Swasthya Mitra cadre?

How long have you been working as a Swasthya Mitra? Can you describe your experience, including the nature of your work and the impact you've had on patients?

Work Environment and Support

Can you describe your interactions with the staff at the healthcare facility (e.g., registration counter staff, doctors, nurses, administrators)? How do they support your work?

What challenges or barriers have you encountered in your role? (Consider aspects like salary, patient load, and support from healthcare facility staff.)

What suggestions do you have for overcoming these challenges? (Consider changes at the administration level, within the JEEViKA cadre, or related to your duties.)

Patient Interaction and Follow-up

How would you describe your overall experience with patients and their companions? (Consider conflicts, clarifications of doubts, and counselling.)

Do you follow up with patients you have previously assisted? If so, how do you manage this, and what challenges do you face?

Impact Assessment

What is your perspective on the impact of the services you provide? (Consider the preference of private over government hospitals and patient footfalls.)

Interview Guide for the Swasthya Mitra Project

Doctors/Nurses/Administrators Interview Guide

Awareness and Experience

Are you familiar with the Swasthya Mitra cadre and their role within the hospital?

What challenges do patients face in large government hospitals? (Consider waiting times, guidance, literacy, patient load, and the role of intermediaries.)

Can you share your experiences with patients assisted by Swasthya Mitras?

Benefits and Support

What benefits have you observed from the services provided by Swasthya Mitras?

How do you support Swasthya Mitras in their role?

What improvements or expansions do you suggest for the Swasthya Mitra cadre?

Beneficiaries Interview Guide

Awareness and Service Utilization

Are you aware of the Swasthya Mitra cadre and the services they provide?

Where do you usually seek medical treatment? Have you used the services of a Swasthya Mitra, and what was the process like?

Experience and Challenges

How was your experience with the Swasthya Mitra in your locality? (Consider the services provided, efficiency, and communication skills.)

Did you encounter any challenges while accessing healthcare services through a Swasthya Mitra? (Consider delays or difficulties in contacting them.)

How could these challenges be addressed or improved?

Post-Consultation Experience

Can you describe your experience after the consultation and treatment at the hospital? (Consider follow-up and tracking by the Swasthya Mitra.)
